# SLC34A2 Up-regulation And SLC4A4 Down-regulation Correlates With Invasion, Metastasis, And The MAPK Signaling Pathway In Papillary Thyroid Carcinomas

**DOI:** 10.7150/jca.56730

**Published:** 2021-07-13

**Authors:** Fengyan Huang, Haitao Wang, Juan Xiao, Chunchun Shao, Yong Zhou, Wei Cong, Maosong Gong, Jingfu Sun, Liqun Shan, Zhanyu Hao, Lihua Wang, Shouluan Ding, Zhigang Yu, Jianing Liu, Hongying Jia

**Affiliations:** 1 Department of Epidemiology and Health Statistics, School of Public Health, Cheeloo College of Medicine, Shandong University, Jinan, Shandong 250012, P.R. China; 2 Department of pathology, The Second Hospital, Cheeloo College of Medicine, Shandong University, Jinan, Shandong 250033, P.R. China; 3 Evidence based medicine, The Second Hospital, Cheeloo College of Medicine, Shandong University, Jinan, Shandong 250033, P.R. China; 4 Medical laboratory center, The Second Hospital, Cheeloo College of Medicine, Shandong University, Jinan, Shandong 250033, P.R. China; 5 Department of Thyroid Surgery, The Second Hospital, Cheeloo College of Medicine, Shandong University, Jinan, Shandong 250033, P.R. China; 6 Department of Breast Surgery, The Second Hospital, Cheeloo College of Medicine, Shandong University, Jinan, Shandong 250033, P.R. China

**Keywords:** papillary thyroid carcinoma, SLC transporters, invasion, metastasis, MAPK signaling pathway

## Abstract

Papillary thyroid carcinoma (PTC) is one of the fastest growing endocrine system malignant carcinomas detected over the past decade. Unfortunately, more than 25% of PTC patients are characterized by their aggressiveness and subsequent metastasis; these characteristics usually indicate poor prognosis. Recently, increasing evidence has suggested that solute carrier (SLC) transporters may play a pivotal role in the initiation, invasion and metastasis of human carcinoma. However, the expression and clinicopathological significance of SLC transporters in patients with PTC remains undetermined. In this study, we aimed to elucidate how the differential expression of SLC transporters affects clinicopathological features, as well as determine the possible regulatory signaling pathways involved. Three differentially expressed SLC transporters were screened from the Gene Expression Omnibus (GEO) and The Cancer Genome Atlas (TCGA) database using a bioinformatics approach. The results indicated that high SLC34A2 and low SLC4A4 protein expression exhibited a higher percentage of capsular invasion and extra-thyroid metastasis in patients. Logistic regression analysis showed that high SLC34A2 expression in tumors was identified as an independent risk factor for capsular invasion [odds ratio (OR)=11.400, 95% confidence interval (CI)=1.733-74.995, P=0.011] and extra-thyroid metastasis (OR=4.920, 95%CI=1.234-19.623, P=0.024), while low SLC4A4 expression in tumors was only identified as independent risk factors for extra-thyroid metastasis (OR=8.568, 95%CI =1.186-61.906, P=0.033). Specifically, for tumors with capsular invasion and extra-thyroid metastasis, the protein expression staining of SLC34A2 was markedly enhanced in the cytoplasm of follicular epithelial cells, contrastingly, SLC4A4 expression was notably weakened in the cytomembrane and nucleus. Intriguingly, both high SLC34A2 and low SLC4A4 protein expression were significantly linked to a high urinary iodine concentration in patients with PTC. Mechanistically, compared with adjacent normal thyroids, p-ERK was significantly up-regulated by 17.8% in the invading tumor; p-ERK, p-JNK, and p-P38 were markedly up-regulated by 29.2%, 67.1%, and 38.9% for metastatic tumors, respectively. Importantly, SLC4A4 negatively correlated with p-JNK (r=-0.696, P= 0.004) and p-P38 (r=-0.534, P=0.049). In conclusion, we suggest that up-regulated SLC34A2 (mainly in the cytoplasm) and down-regulated SLC4A4 (mainly in the cytomembrane and nucleus), which might be attributed to excess iodine intake, were closely linked to extra-thyroid metastasis in PTCs. Furthermore, this effect of SLC4A4 may be through the activation of JNK/P38 MAPK signaling pathway. Future *in vivo* and *in vitro* gain- or loss-of-function experiments are needed to verify these findings and further elucidate the deeper molecular mechanisms.

## Introduction

Thyroid cancer is the fastest growing endocrine system malignant cancer in recent decades 1. Papillary thyroid cancer (PTC) is the most predominant of the histopathological types, with 84% accounting for thyroid cancer 1. The majority of patients with PTC possess indolent progression. Unfortunately, more than 25% of patients with PTC are characterized by the disease's aggressiveness and metastatic nature; these characteristics usually indicate a poor prognosis 2, 3. However, the exploration of clinical biomarkers and molecular mechanisms for the invasion and metastasis of PTCs remains insufficient.

Solute carrier (SLC) transporters represent the largest family of transmembrane proteins encoded in the human genome 4. These transporters utilize an electrochemical or ion gradient to facilitate the exchange of various substances, including endogenous or xenobiotic compounds as well as molecules across the biological membranes, such as sugars, nucleotides, amino acids, nutrients, and drugs 5, 6. Recently, a several studies have highlighted that SLC transporters might play a critical role in the initiation, progression, metastasis, and prognosis of human carcinoma 7, 8. For instance, Kikuchi *et al* 9 found that up-regulated SLC37A1 was significantly associated with the venous invasion and liver metastasis of colorectal cancer. Moreover, Ding *et al* 10 indicated that SLC39A7, SLC39A11, and SLC39A14 were significantly linked to favorable prognosis in patients with gastric cancer. El-Ansari *et al* 11 utilized co-operative expression analysis and demonstrated that SLC1A5, SLC7A5, and SLC3A2 were distinctly correlated to both the high proliferation and aggressive nature of the ER+ breast cancer. He *et al* 12 indicated that SLC34A2 might boost the proliferation, cell cycle progression, migration, invasion, and adhesion of PTC cell *in vitro* via PTEN/AKT/FOXO3 signaling pathway. Despite all aforementioned findings, the effect of the expression and clinicopathological significance of SLC transporters on PTCs has not yet been fully elucidated.

Therefore, in this study, we aimed to evaluate the differential expression of SLC transporters and determine their effect on clinicopathological characteristics, further devoted to reveal possible regulatory signaling pathways involved. Undoubtedly, the results will provide a novel insight into expound the deeper mechanism of invasion and metastasis of patients with PTC, and offer a research reference of clinical biomarkers and new therapeutic targets to some extent.

## Materials and Methods

### Study population selected

This study involved patients with thyroid nodules who underwent thyroidectomy at the Department of Thyroid Surgery, The Second Hospital of Shandong University (Jinan, China), from July 2019 to December 2019. The included criteria were as follows: i) patients with initial thyroidectomy; ii) patients were diagnosed as the resectable primary tumors; iii) patients hadn't received previous treatment for the tumor before the surgical excision; iv) recently hadn't received the therapeutic iodine (2 months) nor used of anti-thyroid drugs or thyroid hormone therapy; v) patients hadn't accompanied with kidney or liver dysfunction, or other systemic diseases. After excluding medullary, follicular, and undifferentiated thyroid carcinoma, 48 cases of PTC remained.

The pathohistological type was determined by the pathologist from the Department of Pathology of The Second Hospital of Shandong University. Pathological characteristics, including primary tumor size and location, capsular invasion, extra-thyroid metastasis, complication, and lymph node location [which were evaluated based on the National Comprehensive Carcinoma Network guidelines for thyroid carcinoma recommendation (https://www.nccn.org)], were extracted from the pathological report; particularly, after routine pathological diagnoses, lymph nodes were detected in 43 cases of PTC among 48 patients, and other 5 cases cannot found any lymph nodes. Refer to previously published standards 13, patients with thyroid nodules were divided into two groups in present study according to Thyroid Imaging Reporting and Data System (TIRADS) grading: TIRADS 2, 3, 4a, and 4b were in a group, TIRADS 4c and 5 were in a group. Descriptions of nodule feathers was obtained from the latest ultrasound reports.

This study was reviewed and approved by The Research Ethics committee of The Second Hospital of Shandong University [no. KYLL-2019(KJ)P-0084]. This study was performed in accordance with the ethical standards of the Declaration of Helsinki. Written informed consent was obtained from all patients before using their samples and clinical data.

### Bioinformatics analysis

The mRNA expression profiles of GSE3678, GSE29265, GSE33630, and GSE50901 microarrays in tumor tissues and matched normal thyroid tissues were compared using the Gene Expression Omnibus (http://www.ncbi.nlm.nih.gov/geo/) database (GEO). The screening process is shown in Supplementary Table 1. The raw data were downloaded and analyzed using the “limma” package in R software (version 3.5.1), in which the differentially expressed genes (DEGs) that met the cut-off criteria of an adjusted P < 0.05 and a |log2 Fold-change| of >1.0 were screened. The package “heatmap.2” was used to perform the hierarchical clustering analysis. The screened DEGs were processed with Venn analysis, and intersection genes were considered as overlapping DEGs. The gene expression profiles were based on RNA-seq and relevant clinical data from The Cancer Genome Atlas (TCGA) database. This comprised 512 PTC tumor tissues and 337 normal thyroid tissues, which were downloaded from the cBioPortal for Cancer Genomics (https://www.cbioportal.org/) online platform 14 and used for expression comparison and survival [including disease/progression-free survival (DFS/PFS)] analysis.

### Tissue RNA isolation and real-time PCR (RT-PCR) assay

Total RNA was extracted from fresh frozen specimens using a RNA reagent kit (No. G3013, Servicebio, China) following the manufacturer's instructions. cDNA was obtained using the RT Synthesis reagent Kit (No. G3330, Servicebio, China) according to manufacturer's instructions. RT-PCR was performed with the LightCycler 480 System (Roche Diagnostics, IN, USA). The PCR profile was as follows: the thermocycling conditions were 95°C for 10 min followed by 40 cycles of 95°C for 15 s and 60°C for 60 s. GAPDH was used as a reference gene. The following forward and reverse primers were used for PCR:

SLC34A2, 5'-AGGGCTTATCCACTCACGCTG-3' and 5'-GATGATGAAGACGACGGGAAC-3'; SLC4A4, 5'-AGCCAACAAGTCCAAACCGA-3' and 5'-CTACATACCACGGAAGAGCCAT-3'; SLC25A15, 5'-GCTGCCTGAAGACTTACTCCCA-3' and 5'-TTCTGGCTCTTGGCTATCTTCC-3'; GAPDH, 5'-GGAAGCTTGTCATCAATGGAAATC-3' and 5'-TGATGACCCTTTTGGCTCCC-3'. The relative changes in the gene expression were calculated according to 2^-ΔΔCt^ method for quantification 15.

### Tissue protein extraction and western blotting analysis

The fresh frozen tissues were washed 3 times in PBS and homogenized ice-cold RIPA buffer containing phosphatase inhibitors, phenylmethanesulfonyl fluoride (PMSF), and protease inhibitor cocktail. Protein concentrations were determined by BCA protein quantification kit 16. Proteins were separated by 10% SDS-PAGE with a 4% stacking gel and transferred to a PVDF membrane. The membranes were blocked with 5% skim milk for 1.5 h, followed by incubation overnight at 4°C with primary antibodies against SLC34A2 (dilution 1:1000; D6W2G, CST, US), SLC4A4 (dilution 1:1000; ab187511, Abcam, UK), SLC25A5 (dilution 1:1000; ab228604, Abcam, UK), ERK (dilution 1:500; bsm-33337M, Bioss, China), p-ERK (dilution 1:500; bs-1522R, Bioss, China), JNK (dilution 1:500; E-AB-60070, Elabscience, China), p-JNK (dilution 1:500; BM4380, Boster, China), P38 (dilution 1:500; E-AB-32459, Elabscience, China), p-P38 (dilution 1:500; E-AB-21027, Elabscience, China), β-actin (dilution 1:2000; 17AV0412, Zhongshan Golden Bridge, China). After washing 3 times with TBST (TBS containing 0.1% Tween 20), the membrane was incubated with horseradish peroxidase-conjugated secondary antibody (dilution 1:5000; ZB-2301, Zhongshan Golden Bridge, China). The signal was detected using chemiluminescence reagent (Millipore, cat no. WBKLSO100, Burlington, USA), and the intensity of the integrated optical density of the bands was quantitated using AlphaView SA software. Refer to the published reference standard of the protein expression assessment 3, 11, 12, the quantitative analysis enabled the calculation of the ratio of protein expression in tumor tissue compared to adjacent normal thyroid tissue. If the ratio is >1.00, it was defined as high expression; if the ratio is <1.00, it was defined as low expression; if there was no significant differences in the expression between the tumor and normal thyroid tissues, it was defined as unchanged.

### Immunohistochemical (IHC) staining

The protein expression localization were performed by using the method of IHC as described in our previously studies 17, 18. In brief, the tissues were fixed in 4% paraformaldehyde and embedded in paraffin. The slides were deparaffinized, rehydrated, and treated with 3% hydrogen peroxide for 20 min to inhibit the endogenous peroxidase. The sections were rinsed with distilled water and saturated in phosphate buffered saline for 5 min and then incubated with a 1:10000 dilution of rabbit anti-polyclonal primary antibody overnight at 4 °C. The staining was visualized using DAB solution and counterstained with hematoxylin. The IHC staining was conducted in accordance with the manufacturer's instructions.

### Iodine nutrition status evaluation

The methods of urinary iodine concentration (UIC) detection and quality controls, as well as the water iodine assessment were the same as our previously study 19. According to the recommendations of the World Health Organization, the iodine nutritional status of individual's was categorized into three degrees as follows: i) low UIC (<100 ug/l), iodine-deficient; ii) adaptive UIC (100-299 ug/l), iodine-adequate; and iii) high UIC (≥300 ug/l), iodine-excessive. The water iodine concentrations for shallow groundwater of domicile residential district of Shandong province were consulted. According to the grading standard for water iodine concentration, Shandong was divided into three groups as follows: i) historically iodine-deficient regions (median water iodine concentration <10 ug/l); ii) historically iodine-adaptive regions (median water iodine concentration 10-150 ug/l); and iii) historically iodine-excessive regions (median water iodine concentration >150 ug/l).

### Statistical analysis

Data were entered into the software by two researchers and double cross-checked using EpiData 3.0 (https://www.epidata.dk) for quality control. Descriptions of the basic characteristics are expressed as mean ± standard deviation, or as percentages. For continuous variables, differences between the groups were analyzed using ANOVA, a *t*-test, or a nonparametric analysis of variance test (Kruskal-Wallis H test and Mann-Whitney U test) in accordance with the concrete types of data. McNemar's test was used for self-matching comparative analyses. Chi-squared or fisher's exact probability method was used to differentiate the rates of different groups. Bonferroni's test was used for multiple comparisons followed by a post‑hoc test. Logistic regression analysis was also performed using SPSS. Kaplan-Meier method was conducted to calculate the survival curves. A log-rank test was used to determine differences of the survival rates. Spearman rank test was used for linear correlation analyses. These analyses were all performed by SPSS 22.0 (IBM Corp.) and R software (version 3.5.1) (https://www.r-project.org). Sigma Plot (version 14.0), GraphPad prism (version 5.0), and Photoshop (version 13.0.1) software were utilized for visualization. The P value < 0.05 was regarded as statistically significant.

## Results

### Identification of differentially expressed SLC transporters in PTCs

#### Bioinformatics analysis using public database

**Figure 1:**
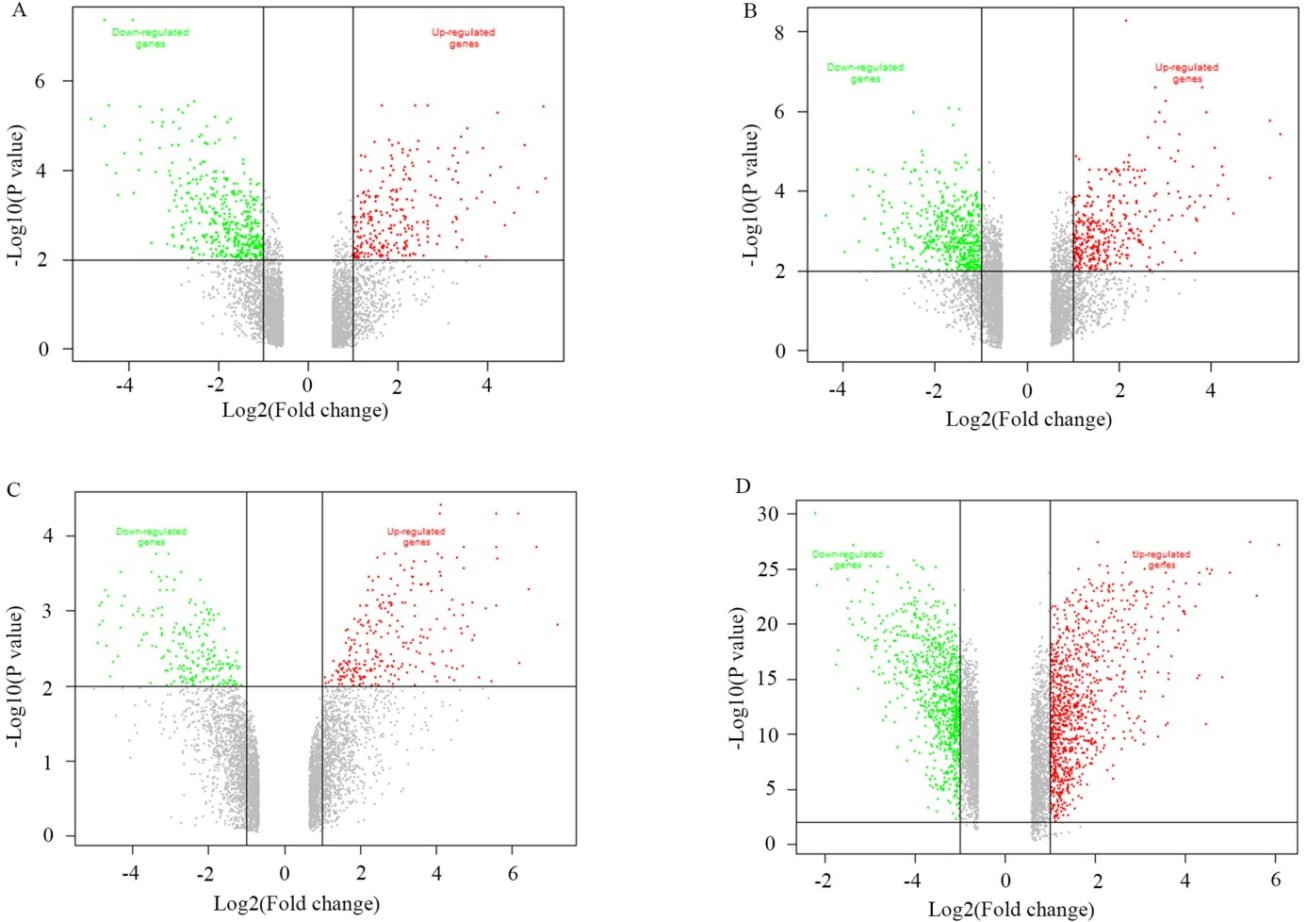
Volcano plot of the genes between tumors with its matched normal thyroids. A-D were namely GSE3678, GSE29265, GSE50901, and GSE33630. P<0.05 and Fold change>2. Red spot stands for up-regulated genes in tumors, green spot stands for down-regulated genes in tumors, while gray spot stands for non-difference.

**Figure 2:**
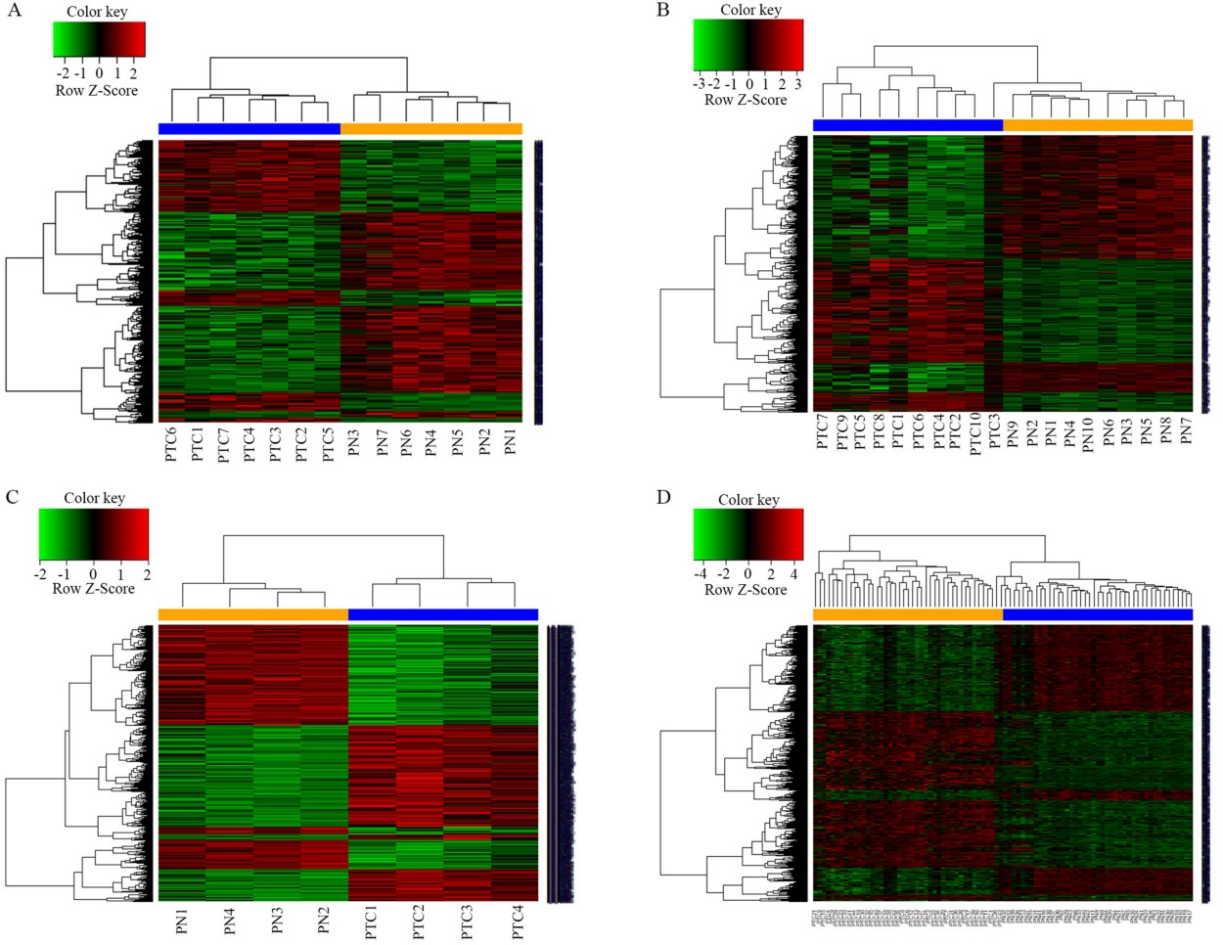
Heatmap of the DEGs between tumors with its matched normal thyroid. A-D were namely GSE3678, GSE29265, GSE50901 and GSE33630. P<0.05; Fold change>2. The depth of the color is correlated with genes expression strength on a linear scale, red stands for up-regulated genes, while green stands for down-regulated genes. The genes dendrogram and samples assignment were shown along the left and the bottom. DEGs, differentially expressed genes.

**Figure 3:**
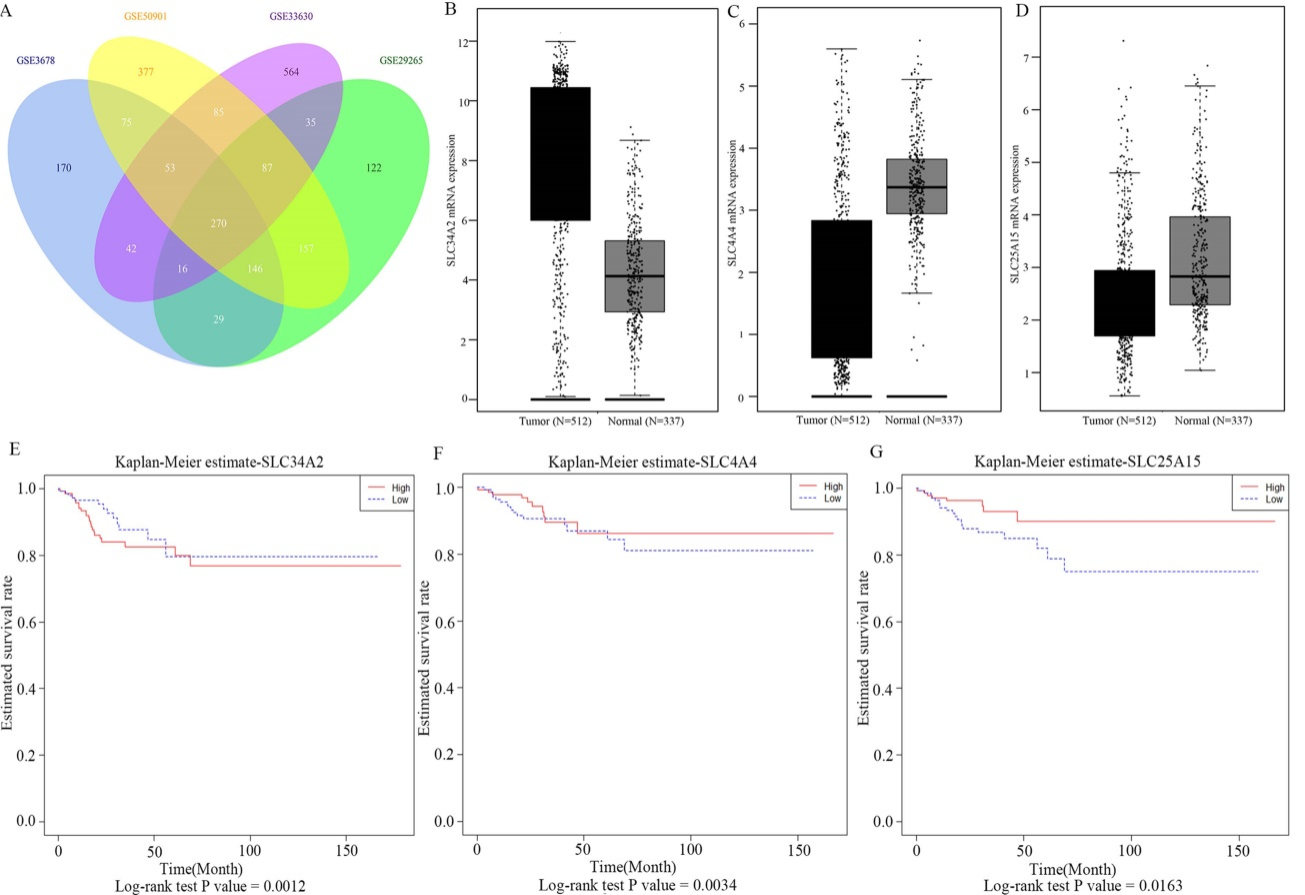
Intersection genes expression and Kaplan-Meier survival analysis based on TCGA clinical cohorts. A was the Venn plot of overlapped DEGs of GSE3678, GSE29265, GSE50901, and GSE33630; B-D were box plot of expression of SLC34A2, SLC4A4, and SLC25A15 in tumor and matched normal thyroid; E-G were the survival curves of SLC34A2, SLC4A4, and SLC25A15, respectively. Black box symbolized the tumor, grey box symbolized the normal. Red line symbolized high expression, blue line symbolized low expression. TCGA: The Cancer Genome Atlas; DEGs: differentially expressed genes.

A total of 1038 DEGs (399 up-regulated and 639 down-regulated) were identified in GSE3678, 1198 DEGs (523 up-regulated and 675 down-regulated) in GSE29265, 1292 DEGs (658 up-regulated and 634 down-regulated) in GSE50901, and 1790 DEGs (974 up-regulated and 816 down-regulated) in GSE33630 (Figure 1). The visualization analysis of hierarchical clusters demonstrated that the above screened DEGs can notably distinguish tumor tissue from the matched normal thyroid in corresponding microarrays datasets (Figure 2). In total, 270 overlapped DEGs (Supplementary Table 2 and Figure 3A) were screened from above four microarray datasets. Among these overlapped DEGs, after excluding any non-coding RNA (likes LncRNA SLC26A4-AS1 20), remaining SLC transporters were all utilized for expression comparison and survival analysis, using the TCGA clinical database. The results revealed that SLC34A2 was mostly overexpressed in tumors when compared with the normal thyroid. Contrastingly, SLC4A4 and SLC25A15 were mostly decreased in tumors when compared with normal thyroids (Figure 3B-D). Furthermore, the higher expression of SLC34A2 and the lower expression of both SLC4A4 and SLC25A15 were all significantly correlated with shorter DFS/PFS (log-rank test P value all < 0.05) of patients of PTC (Figure 3E-G). In other words, SLC34A2 usually acts as factor in unfavorable prognoses, whereas SLC4A4 and SLC25A15 act as the preventive factors and can prolong the progress-free periods of PTCs. Thus, these three genes were all chosen for further analysis.

#### Expression of SLC transporters in both tumor and adjacent normal thyroid

**Figure 4:**
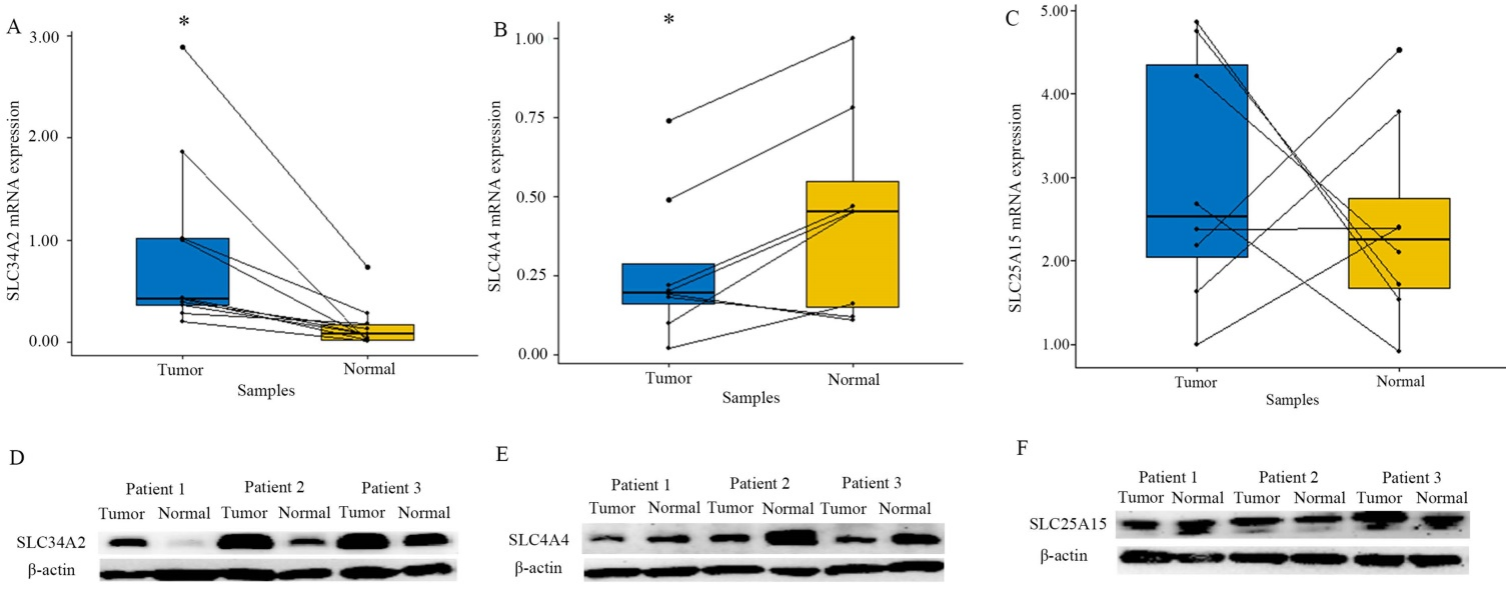
Expression of SLC34A2 (A and D), SLC4A4 (B and E), and SLC25A15 (C and F) in tumor tissues and adjacent normal thyroid tissues. A-C were the mRNA expression analysis by RT-PCR (N=8); D-F were the representative protein bands of western blotting. “*” represented P < 0.05.

To verify the accuracy of the result of the GEO and TCGA database, we utilized RT-PCR experiment to evaluate SLC34A2, SLC4A4, and SLC25A15 expressions in eight cases of pair matched tissue samples. RT-PCR results showed that expression of *SLC34A2* mRNA was drastically up-regulated while *SLC4A4* mRNA was drastically down-regulated in tumor tissue, when compared with the adjacent normal thyroid tissue (P all < 0.05). A similar trend was observed at the protein expression level for both SLC34A2 and SLC4A4, as evidenced by western blotting. Unfortunately, both the mRNA and protein expression levels of SLC25A15 was not remarkably different between tumor and adjacent normal thyroid (Figure 4). Thus, SLC34A2 and SLC4A4 were selected for subsequent analysis.

### Clinicopathological significance of SLC transporters in patients with PTC

#### Comparison analysis

To explore the possible association between the protein expression of SLC34A2 and SLC4A4 regarding clinicopathological features, western blotting was performed on a clinical cross-section consisting of 48 cases of PTCs; including 12 men and 36 women, with a mean age ± SD of 45.2 ± 13.4 years (range 19-68 years). The results of the comparison analysis are displayed in Tables 1 and Table 2. Approximately half of the PTCs (43.8%, 21/48) exhibited a high SLC34A2 protein expression in tumor tissue compared with the adjacent normal thyroid tissue; 90.5% (19/21), 76.2% (16/21), and 76.2% (16/21) of these PTCs with high SLC34A2 exhibited capsular invasion, extra-thyroid metastasis, and high UIC, respectively; the percentage was dramatically higher than that of the unchanged SLC34A2 expression group [54.5% (12/22), 40.9% (9/22), and 31.8% (7/22), P all < 0.05]. Almost one-third of PTCs (33.3%, 16/48) presented a low SLC4A4 protein expression in tumor tissue compared with the adjacent normal thyroid tissue; 93.7% (15/16), 75.0% (12/16), and 81.2% (13/16) of these PTCs with low SLC4A4 presented capsular invasion, extra-thyroid metastasis, and high UIC, respectively, the percentage was significantly higher than that of the unchanged SLC4A4 expression group [54.2% (13/24), 33.3% (8/24), and 37.5% (9/24), P all < 0.05].

#### Logistic regression analysis

As shown in Tables 3 and Table 4, logistic regression analysis indicated that PTCs with a high SLC34A2 protein expression in tumor tissue was identified as the independent predictor for the risk of both capsular invasion [univariate analysis: odds ratio (OR) = 7.917, 95% confidence interval (CI) =1.473-42.538, P=0.016; multivariate analysis: OR=11.400; 95% CI=1.733-74.995, P=0.011)] and extra-thyroid metastasis (univariate analysis: OR=4.622, 95% CI=1.240-17.266, P=0.023; multivariate analysis: OR=4.920, 95% CI =1.234-19.623, P=0.024) in PTCs. While low SLC4A4 protein expression in tumors (univariate analysis: OR=6.000, 95% CI=1.458-24.686, P=0.013; multivariate analysis: OR=8.568, 95% CI=1.186-61.906, P=0.033) was only determined as the independent predictor for the risk of extra-thyroid metastasis of PTCs. This finding indicated that high level of SLC34A2 might be closely associated with the risk of both capsular invasion and extra-thyroid metastasis, while low SLC4A4 was only closely related to the risk of extra-thyroid metastasis.

As shown in Table 5 and Table 6, logistic regression analysis exhibited that PTCs with a high UIC was identified as the independent predictor for the risk of the high SLC34A2 protein expression (univariate analysis: OR = 8.381, 95% CI = 1.770-39.692, P=0.007; multivariate analysis: OR=7.780, 95% CI=1.165-37.488, P=0.011) and the low SLC4A4 protein expression (univariate analysis: OR=17.333, 95% CI=1.902-157.999, P =0.011; multivariate analysis: OR=18.179, 95% CI=1.840-179.554, P=0.013). These findings illustrated that a high UIC might be closely linked to the high SLC34A2 and low SLC4A4 protein expression.

#### Locating analysis

**Figure 5:**
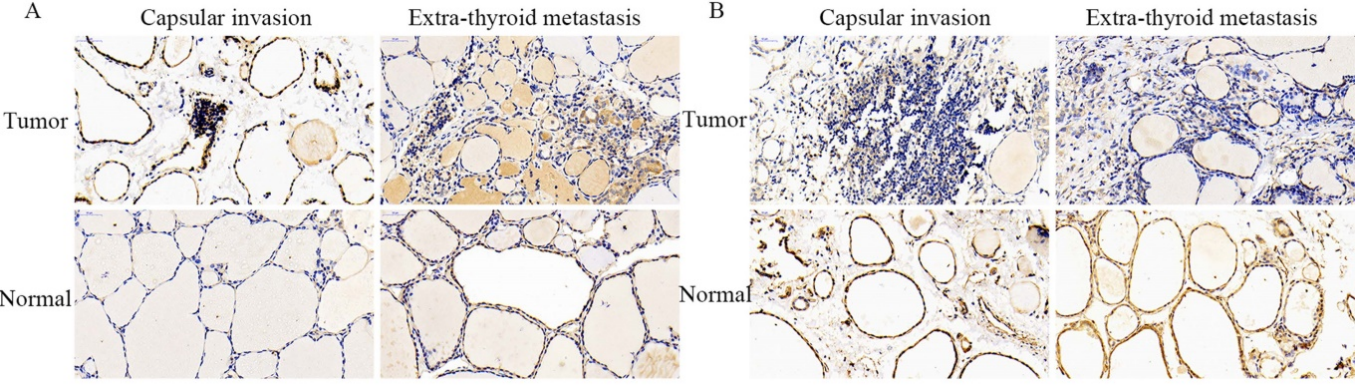
Representative immunohistochemical staining for SLC34A2 and SLC4A4 in tumor tissues and adjacent normal thyroid tissues of PTCs who suffered with capsule invasion and extra-thyroid metastasis, respectively. Scale is 400× for each picture. PTC, papillary thyroid carcinoma.

The representative staining images obtained from IHC assays are shown in Figure 5. Consistent with the above findings, the positive staining intensity of SLC34A2 protein expression was demonstrated to be markedly enhanced in the cytoplasm of follicular epithelial cells of the tumor tissue of PTCs who suffered with capsular invasion and extra-thyroid metastasis, compared to their corresponding adjacent normal thyroid. Contrastingly, the negative staining intensity of SLC4A4 protein expression was notably weak in the cytomembranes and nuclei of follicular epithelial cells of the tumor tissue of PTCs who suffered with capsular invasion and extra-thyroid metastasis, compared to their corresponding adjacent normal thyroid. These results might help to determine the cellular localization of SLC34A2 and SLC4A4 protein expression level in tumor tissue of PTC patients who have undergone capsular invasion and extra-thyroid metastasis.

### Correlation analysis of SLC transporters with MAPK signaling pathway

#### Association between MAPK signaling pathway with capsular invasion and extra-thyroid metastasis

**Figure 6:**
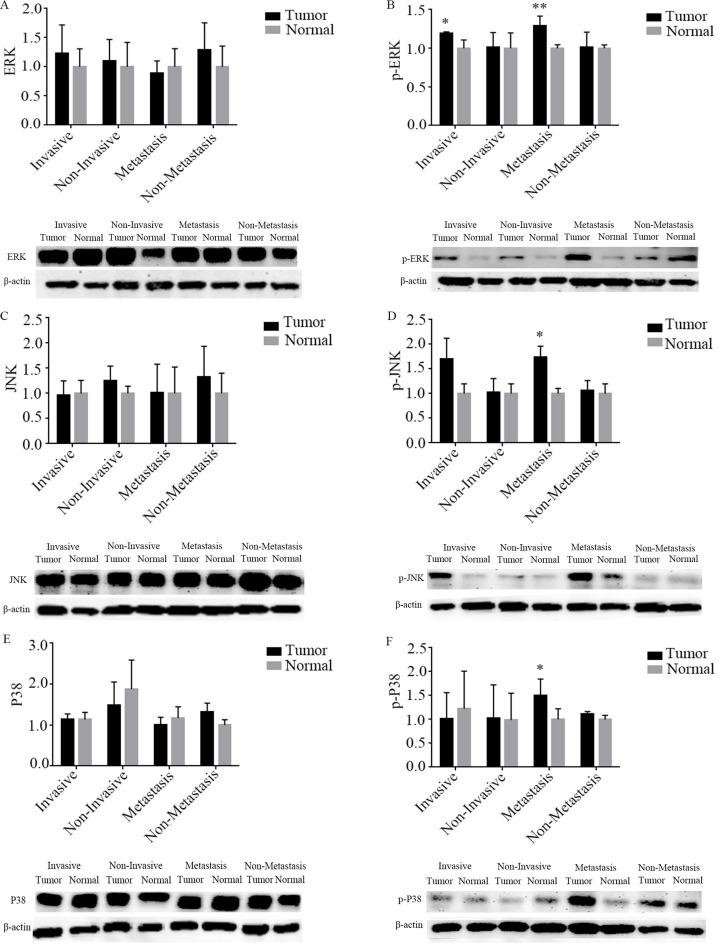
Expression of MAPK signaling pathway in tumor tissues and adjacent normal thyroid of PTCs who suffered with capsule invasion and extra-thyroid metastasis by western blotting. A-F were namely ERK, p-ERK, JNK, p-JNK, P38, and p-P38. “*” represented P < 0.05, “**” represented P < 0.01. PTC, papillary thyroid carcinoma.

As shown in Figure 6, the results of the western blotting showed that p-ERK protein expression was significantly up-regulated by 17.8% and 29.2% for capsular invasion and extra-thyroid metastasis tumors, respectively, when compared to their corresponding adjacent normal thyroid (P all < 0.05). The protein expression of p-JNK was markedly up-regulated by 67.1% for extra-thyroid metastasis tumors, compared to the adjacent normal thyroid (P < 0.05). The protein expression of p-P38 was significantly up-regulated by 38.9% for extra-thyroid metastasis tumors, when compared to the adjacent normal thyroid (P < 0.05). However, no significant differences were observed for the protein expression of p-ERK, p-JNK, and p-P38 between tumor tissues and their corresponding adjacent normal thyroids (P all > 0.05) of the PTCs who cannot undergone any capsular invasion nor extra-thyroid metastasis. Hence, the phosphorylation activation of ERK might be both related to tumor capsular invasion and extra-thyroid metastasis of PTCs, while phosphorylation activation of JNK and P38 might be only related to extra-thyroid metastasis.

#### Correlations between SLC34A2 and SLC4A4 with phosphorylation of ERK, JNK, and P38

**Figure 7:**
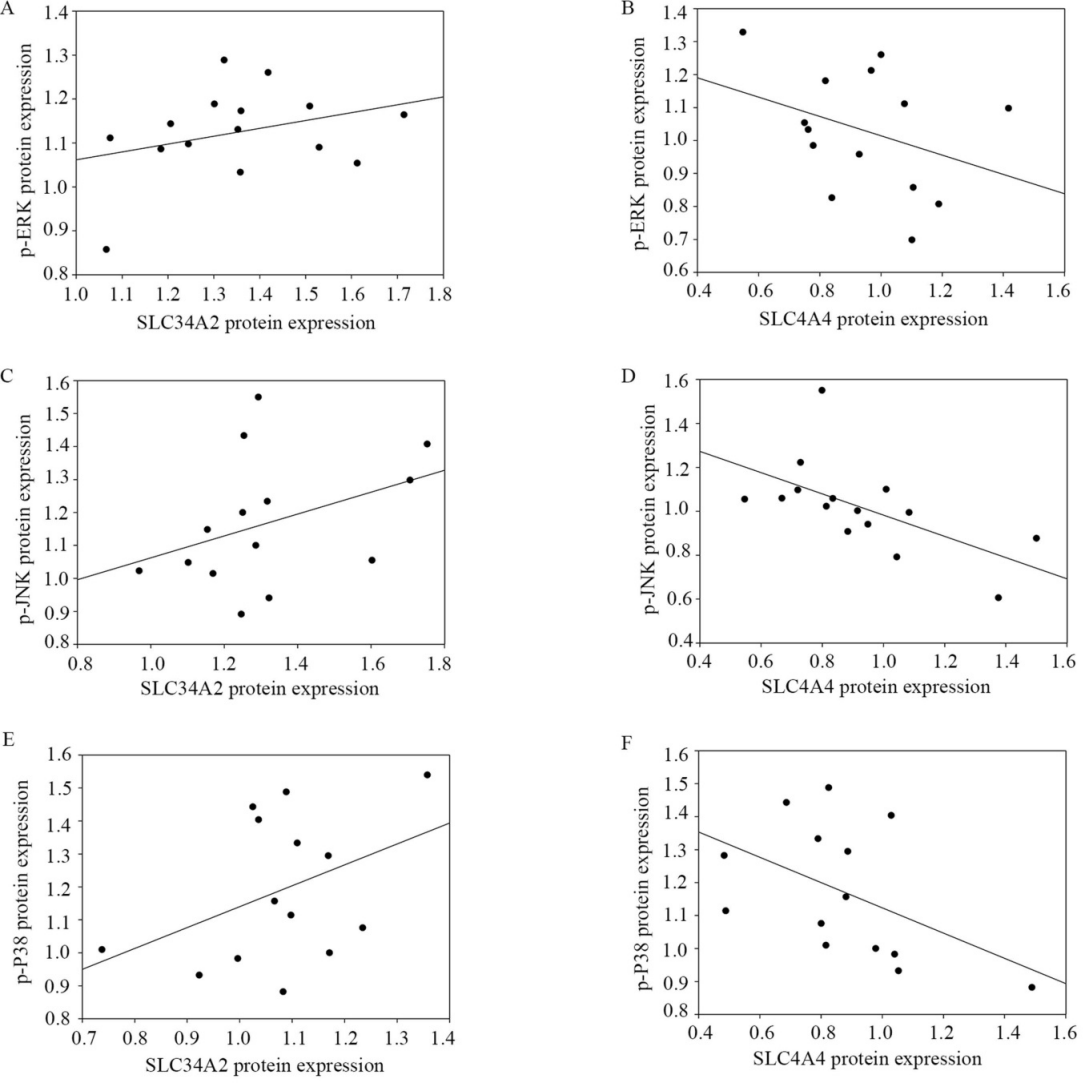
Correlation of SLC34A2 and SLC4A4 expression with MAPK signaling pathway of PTCs. A and B namely the correlation between p-ERK with SLC34A2 (r=0.225, P=0.420) and SLC4A4 (r=-0.354, P=0.215); C and D namely the correlation between p-JNK with SLC34A2 (r=0.437, P=0.118) and SLC4A4 (r=-0.696, P=0.004); E and F namely the correlation between p-P38 with SLC34A2 (r=0.336, P= 0.240) and SLC4A4 (r=-0. 534, P=0.049). PTC, papillary thyroid carcinoma.

Western blotting was used to determine the possible association between SLC34A2 and SLC4A4 protein expression with the p-ERK, p-JNK, and p-P38 protein expression in tumor tissues when compared to the adjacent normal thyroid tissue. As shown in Figure 7, a significantly negative correlation was observed between SLC4A4 protein expression with p-JNK (r=-0.696, P =0.004) and p-P38 (r=-0.534, P =0.049). Unfortunately, no remarkable correlations were observed between the relative expression of SLC34A2 with p-ERK, p-JNK, and p-P38 (P all > 0.05), as well, between SLC4A4 and p-ERK (P > 0.05). These results enable us to speculate that SLC4A4 down-regulation is closely associated with the activation of the phosphorylation of JNK and P38, yet SLC34A2 is irrelevant to the activation of the MAPK signaling pathway.

## Discussion

Generally, SLC transporters are serve as the transmembrane protein that can facilitate the exchange of various substances and molecules across intracellular and extracellular. Thereby, some SLC transporters were regarded as potential therapeutic targets for certain tumors, such as in the case of the sodium/iodine symporter, the norepinephrine transporter, and the monocarboxylate transporter 21. Increasing evidence suggests that SLC transporters not only function to transport the endogenous and xenobiotic compounds, but also may play a pivotal role in the initiation and progression of thyroid carcinomas 22, 23. However, the clinicopathological significance of the SLC transporter expression in patients of PTC, as well as its involvement in the regulation mechanism of signaling pathways have not yet been completely elucidated. In this study, through bioinformatics analysis from the GEO database, and combined with mining results of TCGA clinical cohort database, the gene, namely SLC34A2 (mediate phosphate absorption), SLC4A4 (transport sodium bicarbonate), and SLC25A15 (exchange ornithine and citrulline), were established as the preliminary candidate differentially expressed SLC transporters for further experiment research. And the validation results of RT-PCR experiment demonstrated that the SLC34A2 mRNA was drastically up-regulated while SLC4A4 mRNA was significantly down-regulated; however, SLC25A15 mRNA showed no significant differences between tumor and normal tissue; furthermore, the trends of protein expression were the same as those of mRNA levels. It should be noted that SLC34A2 was markedly overexpressed both in thyroid cancer and gastric cancer, whereas SLC4A4 knockdown promote proliferation, migration, and invasion of MDA-MB-231 breast cancer cells and LS174 colon cancer cells 12, 22. Together, these information sufficient supported that SLC34A2 and SLC4A4 were the true differentially expressed SLC transporters of PTCs.

Notably, a characteristic of this study is the fact that the clinicopathological significance of SLC34A2 and SLC4A2 protein expression for patients with PTC were preliminary evaluated. The results of western blotting revealed that the percentage of capsular invasion and extra-thyroid metastasis were markedly higher in patients with high levels of SLC34A2 (90.5% vs 54.5% and 76.2% vs 40.9%, P all < 0.05) and low levels of SLC4A4 (93.7% vs 54.2% and 75.0% vs 33.3%, P all < 0.05), and high SLC34A2 protein expression can serve as a risk predictor both for capsular invasion and extra-thyroid metastasis while low SLC4A4 protein expression can only serve as a risk predictor for extra-thyroid metastasis. It was reported that the abnormal expression of SLC transporters might be significantly linked to both the local invasion and distant metastases presented in patients with thyroid carcinoma 23, 24. Thus, it can be inferred that high level of SLC34A2 both closely interrelated capsular invasion risk and extra-thyroid metastasis risk, whereas low SLC4A4 level was only closely related to the risk of extra-thyroid metastasis. Moreover, localization results illustrated that positive staining of the SLC34A2 protein was mainly enhanced in the cytoplasm of thyroid follicular epithelial cells in tumor of PTCs who undergone capsular invasion and extra-thyroid metastasis, while SLC4A4 protein was found to be weak in the cytomembrane and nuclei. These results commonly indicated SLC34A2 protein overexpression might be ascribed for the SLC34A2 expression enhancement in cytoplasm; in contrast, low SLC4A4 expression might be attributed to poor SLC4A4 expression both in cytomembrane and nucleus. Taken together, it should be speculated that the enhancement of SLC34A2 protein expression in the cytoplasm might be contribute to the risk of both capsular invasion and extra-thyroid metastasis, whereas the reduction of SLC4A4 protein expression in the cytomembrane and nucleus might be only closely related to the risk of extra-thyroid metastasis.

An incidental discovery of this study is that the expression of both SLC34A2 and SLC4A4 proteins might be disturbed by the individual's excessive iodine nutrition intakes. Comparative results exhibited that the proportion of high levels of UIC (≥ 300 ug/L) in patients with high SLC34A2 protein expression might be higher than that in PTC patients with unchanged expression (76.2% *vs* 31.8%, P < 0.05); meanwhile, the proportion of high levels of UIC (≥300 ug/L) in patients with low SLC4A4 protein expression was higher than that in patients with unchanged expression (81.2% *vs* 37.5%, P < 0.05). Logistic regression analysis results also illustrated a high UIC might serves as the independent predictor for the risk of high SLC34A2 and low SLC4A4 of PTC patients. It is also well known that UIC is a widely accepted evaluate indicator of individual's iodine nutritional intakes, because more than 90% of ingested iodine is excreted through the urine. Furthermore, previous study demonstrated that high iodine stimulation (supplemented dose, 7.3 mg/L) can markedly perturb the mRNA and protein expression of SLC transporters (SLC5A5 and SLC26A4) in thyroid tissues of Wistar rats, in comparison to control group 25. Combined with the results of our previous research that high iodine closely associated with the risk of capsule invasion and thyroid metastasis of PTCs 19. Therefore, these data together supported a speculation that excess iodine intakes might through up-regulated of SLC34A2 and down-regulated of SLC4A4, contributed to capsule invasion and extra-thyroid metastasis of PTCs. However, further functional experiments needed to verify of this speculation.

With regard to mechanisms, on the one hand, the activation of MAPK signaling pathway has been proven to be the indispensable factor in the occurrence and progression of PTCs 26, 27. However, on the other hand, previous reports have suggested that the suppression of SLC34A2 can repress tumor cell growth, migration, and metastasis of gastric cancer through inhibition of phosphorylated ERK 28. Activation of SLC4A4 via inhibited the phosphorylated of P38 to limit the cell death in ischemia-reperfusion injury rat hearts 29; however, inhibition of JNK (SP600125, 10 uM) cannot effect SLC4A4 protein abundance in cortical astrocytes cells 30. Notably, ERK, JNK, and P38 are together compose the MAPK signaling pathway 31. Therefore, it can be hypothesized that the MAPK signaling pathway might act as a pivotal downstream (rather than upstream) signaling pathway for SLC34A2 and SLC4A4, to promote the tumor progression of PTCs. Based on this hypothesis, we determined to detect the key molecules of the MAPK signaling pathway by western blotting to explore the possible regulatory mechanisms for SLC34A2 and SLC4A4 that underwent capsular invasion and extra-thyroid metastasis. Our results demonstrated that p-ERK was significantly up-regulated by 17.8% in tumors for capsular invasion, while p-ERK, p-JNK, and p-P38 were significantly up-regulated by 29.2%, 67.1%, and 38.9%, in tumors that underwent extra-thyroid metastasis, respectively. Thus, the activation of p-ERK might contribute to both capsular invasion and extra-thyroid metastasis; however, the activation of p-JNK and p-P38 might solely responsible for extra-thyroid metastasis. In one aspect, the activation of ERK could via the acceleration of epithelial-mesenchymal transition (EMT) 32 to provoke PTC cells (TPC-1 and K1) invasiveness and migration, which was previously detected by CCK8 assay 33, 34. However, in other aspect, both P38 (inhibitor, SB203580) and JNK (inhibitor, SP600125) inhibition can generate intracellular reactive oxygen species (ROS) 35, and blocking apoptosis 36 and stimulating stroma-secreted inflammation 37, which could commonly promote the tumor cell metastasis of PTCs 38, 39. Therefore, we speculated that p-ERK activation might through accelerate the EMT and contribute to the capsular invasion and extra-thyroid metastasis of PTCs, whereas p-JNK and p-P38 might be activated through the generation of intracellular ROS, blocked apoptosis, and stimulated inflammation, which together contributed to the extra-thyroid metastasis of PTCs.

Furthermore, linear correlation analysis were utilized to evaluate the association between the MAPK signaling pathway with SLC34A2 and SLC4A4. The results showed that only SLC4A4 was significantly negatively correlated with p-JNK (r=-0.696, P < 0.05) and p-P38 (r=-0.534, P < 0.05). Together with aforementioned mentioned hypothesis that MAPK signaling pathway may serve as a pivotal downstream signal for SLC4A4 to provoke PTC tumor progression. Therefore, it should be inferred that low SLC4A4, via the activation of JNK/P38 MAPK signaling pathway, contribute to the risk of extra-thyroid metastasis of PTCs. However, our results also illustrated that the MAPK signaling pathway does not the significant downstream that correlate with SLC34A2. Therefore, we can infer that SLC34A2 contributed to the risk of capsular invasion and extra-thyroid metastasis not via MAPK signaling pathway, that process might involve in other significantly regulatory pathways, such as AKT 40, NF-κB 41, JAK/STAT 42, and Wnt/β-Catenin 43.

There are some limitations which should be considered when interpreting the results of this study. First, the conclusion would have been more powerful if multiple centers with larger sample sizes were used as the prospective cohort. Second, this was a preliminary exploration of the distribution of gene expression based on clinical cross-sections with small sample sizes, and present can only aid in drawing the possible correlations between SLC34A2 and SLC4A4 with invasion, metastasis, and excess iodine intake of PTCs; subsequent efforts should involve in a series of *in vivo* and *in vitro* gain- or loss-of-function experimental assays (K1 and TPC-1, and xenograft tumor in nude mice) 44 to reveal the upstream and downstream and further explore deeper mechanisms. Lastly, refer to the published experiment design (Wang et al. 45 and Liu et al. 46), considered the advantages of each experiment and the purpose of each step were differences, we only performed western blotting experiment on all of cases and made statistical analysis conclusions, and did not perform RT-PCR and IHC for all 48 cases. Furthermore, our results only supported that SLC34A2 contributed to capsular invasion and extra-thyroid metastasis not via the MAPK signaling pathway, but the possible pathway functioning downstream of SLC34A2 cannot be conclude currently. Despite all these limitations, this study still clarify the clinicopathological significance of differentially expressed SLC transporters and explored possible regulatory signaling pathway which be involved, to some extent. Future work should emphasize *in vivo* and *in vitro* gain- or loss-of-function experimental to validate these findings and further clarify which the major signaling pathway is regulated by SLC34A2 in tumor progression of PTCs.

In conclusion, SLC34A2 (mainly in cytoplasm) up-regulated may contribute to the increased risk of both capsular invasion and extra-thyroid metastasis of PTCs, but this effects may not via the MAPK signaling pathway; whereas SLC4A4 (mainly in the cytomembrane and nucleus) down-regulated might contribute to the risk of extra-thyroid metastasis of PTCs, and this effects through the activation of JNK/P38 MAPK signaling pathway. Furthermore, high SLC34A2 and low SLC4A4, might be all attributable to excessive iodine intakes of individuals. Undoubtedly, the results will provide a new perspective for the research of the role of SLC transporters in invasion and metastasis of PTCs, and more beneficial for deeper molecular mechanism exploration, clinical biomarker identification, and novel therapeutic target discoveries.

## Supplementary Material

Supplementary tables.

## Figures and Tables

**Table 1:** Association between the expression of SLC34A2 with clinicopathological characteristics of PTC patients (mean ± SD, n, %)

Characteristics	SLC34A2			t/χ2 value	P value
	Low	Unchanged	High		
Number^ a^	5 (10.4)	22 (45.8)	21 (43.8)		
Age (years)^ b^	38.6±10.2	48.9±11.7	42.8±15.2	1.825	0.173
Gender^ a,c^				3.221	0.200
Female	2 (40.0)	17 (77.3)	17 (81.0)		
Male	3 (60.0)	5 (22.7)	4 (19.0)		
BMI (Kg/m^2^)^ a,c^				0.097	0.952
<24.0	2 (40.0)	10 (45.5)	10 (47.6)		
≥24.0	3 (60.0)	12 (54.5)	11 (52.4)		
BP (mm/Hg)^ b^					
SBP	78.1±10.7	79.3±7.3	76.9±10.1	1.045	0.360
DBP	132.4±7.7	130.2±12.1	128.2±12.3	0.316	0.730
Water iodine^ a,d^				3.002	0.603
≥150μg/L	1 (20.0)	2 (9.1)	6 (28.6)		
10-150μg/L	3 (60.0)	14 (63.6)	10 (47.6)		
<10μg/L	1 (20.0)	6 (27.3)	5 (23.8)		
Ultrasound					
TIRADS grade^ a,c^				2.157	0.340
≥4c	1 (20.0)	12 (54.5)	11 (52.4)		
≤4b	4 (80.0)	10 (45.5)	10 (47.6)		
Multifocality^ a,c^				0.279	0.870
Yes	2 (40.0)	9 (40.9)	7 (33.3)		
No	3 (60.0)	13 (59.1)	14 (66.7)		
Pathology					
Tumor size^ a,c^				3.263	0.196
>1.0cm	1 (20.0)	8 (36.4)	12 (57.1)		
≤1.0cm	4 (80.0)	14 (63.6)	9 (42.9)		
Capsular invasion ^a.c,e^				7.543	0.023
Yes	4 (80.0)	12 (54.5)	19 (90.5)		
No	1 (20.0)	10 (45.5)	2 (9.5)		
Tumor location^ a,c^				0.245	0.885
Bilaterally	2 (40.0)	7 (31.8)	6 (28.6)		
Unilaterality	3 (60.0)	15 (68.2)	15 (71.4)		
Extra-thyroid metastasis ^a,c,e^				8.385	0.015
Yes	1 (20.0)	9 (40.9)	16 (76.2)		
No	4 (80.0)	13 (59.1)	5 (23.8)		
Complication ^a,c^				0.619	0.734
Yes	3 (60.0)	17 (77.3)	16 (76.2)		
No	2 (40.0)	5 (22.7)	5 (23.8)		
Lymph nodes location ^a,d^				5.792	0.177
Central district	4 (80.0)	19 (86.4)	12 (57.1)		
Non-central district	1 (20.0)	1 (4.5)	6 (28.6)		
No lymph nodes	0 (0.0)	2 (9.1)	3 (14.3)		
UIC (μg/L)^ a,d,e^				9.419	0.034
<100	1 (20.0)	4 (18.2)	2 (9.5)		
100-299	2 (40.0)	11 (50.0)	3 (14.3)		
≥300	2 (40.0)	7 (31.8)	16 (76.2)		

^a^ The numbers (percentage contribution) are listed for each category ^b^ Values are displayed as the mean ± SD ^c^ Correction for continuity ^d^ Fisher's exact probability ^e^ Bonferroni's test shows significantly differences (P <0.05) between high SLC34A2 and unchanged groups in multiple comparisons using χ^2^ analysis followed by a post‑hoc test PTC, papillary thyroid carcinoma; BMI, body mass index; BP, blood pressure; SBP, systolic blood pressure; DBP, diastolic blood pressure; TIRADS, Thyroid Imaging Reporting and Data System. UIC, urinary iodine concentration.

**Table 2:** Association between the expression of SLC4A4 with clinicopathological characteristics of PTC patients (mean ± SD, n, %)

Characteristics	SLC4A4			t/χ2 value	P value
	Low	Unchanged	High		
Number^ a^	16 (33.3)	24 (50.0)	8 (16.7)		
Age (years)^ b^	38.7±13.7	48.5±12.2	47.9±13.8	2.955	0.062
Gender^ a,c^				1.897	0.387
Female	11 (68.7)	20 (83.3)	5 (62.5)		
Male	5 (31.3)	4 (16.7)	3 (37.5)		
BMI (Kg/m^2^)^ a,c^				0.338	0.844
<24.0	8 (50.0)	11 (45.8)	3 (37.5)		
≥24.0	8 (50.0)	13 (54.2)	5 (62.5)		
BP (mm/Hg)^ b^					
SBP	78.7±9.1	78.7±7.5	78.1±10.7	0.016	0.984
DBP	130.3±12.3	128.5±11.6	131.4±12.1	0.227	0.798
Water iodine^ a,d^				3.558	0.511
≥150μg/L	5 (31.2)	3 (12.5)	1 (12.5)		
10-150μg/L	8 (50.0)	13 (54.2)	6 (75.0)		
<10μg/L	3 (18.8)	8 (33.3)	1 (12.5)		
Ultrasound					
TIRADS grade^ a,c^				0.672	0.715
≥4c	8 (50.0)	11 (45.8)	5 (62.5)		
≤4b	8 (50.0)	13 (54.2)	3 (37.5)		
Multifocality^ a,c^				0.790	0.674
Yes	5 (31.3)	9 (37.5)	4 (50.0)		
No	11 (68.7)	15 (62.5)	4 (50.0)		
Pathology					
Tumor size^ a,c^				0.168	0.919
>1.0cm	7 (43.7)	10 (41.7)	4 (50.0)		
≤1.0cm	9 (56.3)	14 (58.3)	4 (50.0)		
Capsular invasion ^a,c,e^				9.458	0.009
Yes	15 (93.7)	13 (54.2)	7 (87.5)		
No	1 (6.3)	11 (45.8)	1 (12.5)		
Tumor location^ a,c^				1.842	0.398
Bilaterally	3 (18.7)	9 (37.5)	3 (37.5)		
Unilaterality	13 (81.3)	15 (62.5)	5 (62.5)		
Extra-thyroid metastasis ^a,c,e^				8.664	0.013
Yes	12 (75.0)	8 (33.3)	6 (75.0)		
No	4 (25.0)	16 (66.7)	2 (25.0)		
Complication^ a,c^				2.377	0.305
Yes	14 (87.5)	16 (66.7)	6 (75.0)		
No	2 (12.5)	8 (33.3)	2 (25.0)		
Lymph nodes location^ a,d^				1.716	0.852
Central district	11 (68.8)	18 (75.0)	6 (75.0)		
Non-central district	3 (18.7)	3 (12.5)	2 (25.0)		
No lymph nodes	2 (12.5)	3 (12.5)	0 (0.0)		
UIC (μg/L)^ a,d,e^				10.713	0.019
<100	2 (12.5)	3 (12.5)	2 (25.0)		
100-299	1 (6.3)	12 (50.0)	3 (37.5)		
≥300	13 (81.2)	9 (37.5)	3 (37.5)		

^a^ The numbers (percentage contribution) are listed for each category ^b^ Values are displayed as the mean ± SD ^c^ Correction for continuity ^d^ Fisher's exact probability ^e^ Bonferroni's test shows significantly differences (P <0.05) between low SLC4A4 and unchanged groups in multiple comparisons using χ^2^ analysis followed by a post‑hoc test PTC, papillary thyroid carcinoma; BMI, body mass index; BP, blood pressure; SBP, systolic blood pressure; DBP, diastolic blood pressure; TIRADS, Thyroid Imaging Reporting and Data System. UIC, urinary iodine concentration.

**Table 3:** Logistic regression analysis for predictors of the capsular invasion risk of PTC patients

Expression	Univariate analysis			Multivariate analysis		
	OR	95%CI	P value	OR	95%CI	P value
SLC34A2						
Unchanged	1.00			1.00		
High	7.917	1.473-42.538	0.016	11.400	1.733-74.995	0.011
Low	3.333	0.319-34.830	0.315	5.417	0.280-104.874	0.264
SLC4A4						
Unchanged	1.00			1.00		
High	5.923	0.628-55.853	0.120	0.360	0.015-8.418	0.525
Low	12.692	1.438-112.019	0.022	7.010	0.581-84.557	0.125

Adjusted potential covariates for multivariate analysis included age, gender, and BMI; PTC, papillary thyroid carcinoma; OR, odds ratio; CI, confidence interval; BMI, body mass index.

**Table 4:** Logistic regression analysis for predictors of the extra-thyroid metastasis risk of PTC patients

Expression	Univariate analysis			Multivariate analysis		
	OR	95%CI	P value	OR	95%CI	P value
SLC34A2						
Unchanged	1.00			1.00		
High	4.622	1.240-17.226	0.023	4.920	1.234-19.623	0.024
Low	0.361	0.034-3.788	0.396	14.465	1.136-184.116	0.040
SLC4A4						
Unchanged	1.00			1.00		
High	6.000	0.981-36.715	0.053	1.014	0.124-8.302	0.990
Low	6.000	1.458-24.686	0.013	8.568	1.186-61.906	0.033

Adjusted potential covariates for multivariate analysis included age, gender, and BMI; PTC, papillary thyroid carcinoma; OR, odds ratio; CI, confidence interval; BMI, body mass index

**Table 5:** Logistic regression analysis of high UIC for high SLC34A2 expression risk of PTC patients

UIC, µg/L	Univariate analysis			Multivariate analysis		
	OR	95%CI	P value	OR	95%CI	P value
100-299	1.00			1.00		
<100	4.571	0.673-31.048	0.120	4.405	0.641-30.284	0.132
≥300	8.381	1.770-39.692	0.007	7.780	1.165-37.488	0.011

Adjusted potential covariates for multivariate analysis included age and gender; PTC, papillary thyroid carcinoma; OR, odds ratio; CI, confidence interval; UIC, urinary iodine concentration.

**Table 6:** Logistic regression analysis of high UIC for low SLC4A4 expression risk of PTC patients

UIC, µg/L	Univariate analysis			Multivariate analysis		
	OR	95%CI	P value	OR	95%CI	P value
100-299	1.00			1.00		
<100	2.167	0.299-15.705	0.444	2.338	0.292-18.746	0.424
≥300	17.333	1.902-157.999	0.011	18.179	1.840-179.554	0.013

Adjusted potential covariates for multivariate analysis included age and gender; PTC, papillary thyroid carcinoma; OR, odds ratio; CI, confidence interval; UIC, urinary iodine concentration.

## Abbreviations

PTC: papillary thyroid carcinoma
SLC: solute carrier
GEO: Gene Expression Omnibus
TCGA: The Cancer Genome Atlas
DEGs: differentially expressed genes
RT-PCR: real-time polymerase chain reaction
ROS: reactive oxygen species
EMT: endothelial-mesenchymal transition
UIC: urinary iodine concentration
IHC: immunohistochemical
TIRADS: Thyroid Imaging Reporting and Data System
