# Supplementary material for: SLC34A2 Up-regulation And SLC4A4 Down-regulation Correlates With Invasion, Metastasis, And The MAPK Signaling Pathway In Papillary Thyroid Carcinomas
Source: J Cancer. 2021 Jul 13;12(18):5439–53. doi: 10.7150/jca.56730 (PMC8364650; doi:10.7150/jca.56730)
Supplement: Supplementary file 1 — Supplementary tables. [file jcav12p5439s1.pdf]

Supplementary Materials

Supplementary Table 1. The screening results of microarrays from GEO database

Series	Platform	Tumor [a]	Normal	Organism	Update data	Country
GSE3678	GPL570	N=7	N=7	Homo sapiens	Mar-25-2019	USA
GSE29265	GPL570	N=10	N=10	Homo sapiens	Mar-25-2019	Belgium
GSE33630	GPL570	N=44	N=44	Homo sapiens	Mar-25-2019	Belgium
GSE50901	GPL13607	N=4	N=4	Homo sapiens	Nov-27-2018	Brazil

[a] Histopathological type of tumor was the papillary thyroid carcinoma. GEO: Gene Expression Omnibus.

Supplementary Table 2. The overlapped DEGs

Gene symbol
ZMAT4, ZFPM2, ZCCHC12, XPR1, XKRX, WSCD2, WFS1, VLDLR, UNC5CL, UNC5B, UBE2QL1, TYMS, TUSC3, TTC39B, TRIM58, TPPP, TPO, TPD52L1, TNS3, TNFRSF11B, TMPRSS4, TMEM171, TMEM139, TMC6, TLE4, TLE1, TIAM1, TGFBR1, TFPI, TFF3, TFCP2L1,TCF7L1, TCEAL2, TC2N, TACSTD2, SYNM, SYNE1, STXBP5L, STK32A, STARD13, SPX, SPINT1, SORBS2, SMAD9, SLC4A4, SLC34A2, SLC26A4-AS1, SLC25A15, SIPA1L2, SHANK2, SFTPB, SFRP1, SERPINA1, SDPR, SDC4, SCUBE3, SCEL, SCARA5, SAMD5, S100A1, RYR2, RYR1, RXRG, RUNX2, RNF150, RELN, RASSF9, RASSF6, RAP1GAP, RAB27A, QPCT, PTPRE, PSD3, PRR15, PROS1, PPARGC1A, POU2F3, PMEPA1, PLXNC1, PLXDC1, PLEKHG4B, PLCH1, PLAG1, PLA2R1, PLA2G7, PKHD1L1, PID1, PGM5, PFKFB2, PDZK1IP1, PDLIM4, PDE5A, PBX4, PAPSS2, OTOS, OGDHL, OCA2, NRP2, NRCAM, NPC2, NOV, NOD1, NGEF, NFE2L3, NCAM1, MYEF2, MT1M, MRO, MRC2, MPZL2, MPPED2, MMRN1, MLLT11, MINA, MFAP4, METTL7B, MET, MEGF9, MCTP2, MAMLD1, MAFB, LRRK2, LRRC2, LRP4, LRP1B, LRIG1, LPAR5, LPAR1, LONRF2, LMOD1, LIPH, LINGO2, LIFR, LGALS3, LEMD1, LAYN, LAMB3, KLK7, KLK10, KLHDC8A, KIT, KIAA1324, KCNJ2, KCNIP4, ITPR1, ITGA2, IRS1, IPCEF1, IP6K3, IGSF10, IGFL2, IGFBPL1, IGF2BP2, IER5L, ID4, HMGA2, HLF, HGD, HEY2, HBB, GSTM3, GPR98, GPR125, GPM6A, GOLT1A, GLT8D2, GJB6, GJB3, GHR, GGCT, GDF15, GALNT7, GALE, GABRB2, FRMD3, FOXQ1, FOXP2, FN1, FMOD, FHL1, FCGBP, FBLN7, FAM20A, FAM167A, FABP4, ETV5, ERBB3, EPS8, EPPK1, EPHA4, EPHA3, ENTPD1, ELMO1, EFEMP1, DUSP6, DUSP4, DTX4, DPT, DPP4, DOCK9, DLG2, DIRAS2, DIO1, DGKI, DDB2, CYP1B1, CXCL12, CWH43, CTSH, CTH, CST6, CSGALNACT1, CRABP1, CORO2A, COMP, COL8A2, COL8A1, COL13A1, CLMN, CLDN10, CLDN1, CLCNKB, CKS2, CITED2, CITED1, CHRDL1, CHI3L1, CFI, CFD, CENPJ, CDON, CDH6, CDH3, CDH16, CD55, CCL21, CCDC85A, CCDC146, CAMK2N1, CA4, C8orf48, C4orf48, C3orf55, C1QTNF7, C19orf33, C11orf74, BLNK, BID, BCL2, AVPR1A, ATP2C2, ATP11A, ARMCX3, ARHGAP24, AOX1, ANKS1B, ANK2, ANGPTL1, AKR1C1, AIF1L, AHNAK2, AGTR1, AGR3, AGR2, AGPAT4, ADH1B, ACACB, ABI3BP, ABCC3, ABCA8

DEGs: differentially expressed genes.
